# Lactate regulation may be a key factor in the protection of the intestinal barrier in sepsis under high-altitude hypoxic and hypobaric conditions

**DOI:** 10.1186/s13054-025-05793-x

**Published:** 2025-12-08

**Authors:** Ruixuan Wang, Cen Wen, Qian Lei, Si Zeng

**Affiliations:** 1 Department of Anesthesiology, School of Medicine, Sichuan Provincial People’s Hospital, University of Electronic Science and Technology of China, Chengdu, 610072 Sichuan PR China; 2 School of Medical and Life Sciences, Chengdu University of Traditional Chinese Medicine, Chengdu, 610072 Sichuan PR China

Dear editors,

We read with great interest the recent correspondence by You et al. [1] published in Critical Care, which highlighted the critical regulatory role of intestinal lactate in epithelial renewal during sepsis. Using a cecal ligation and puncture (CLP) model, the authors demonstrated decreased lactate levels in intestinal tissue but increased levels in serum, accompanied by altered expression of pyruvate dehydrogenase (PDH) and lactate dehydrogenase (LDH). Continuous enteral lactate supplementation improved intestinal length, villus height, and survival, underscoring that lactate functions not only as a metabolic substrate but also as a signaling molecule promoting mucosal repair.

This concept is closely aligned with our earlier clinical findings. Clinical research has demonstrated that high-altitude hypoxia adaptation helps maintain intraoperative glucose metabolic homeostasis in patients and reduces postoperative pulmonary complications in high-altitude patients through lactate regulation [2]. To expand on this concept, we investigated the effects of chronic high-altitude hypoxic adaptation (HA) on intestinal injury in a lipopolysaccharide (LPS)-induced sepsis rat model. Specifically, lipopolysaccharide (L2630-100 mg, Sigma-Aldrich, USA) extracted from Escherichia coli was administered intraperitoneally at a dose of 10 mg/kg to induce sepsis. To align with the lactate-centered hypothesis, arterial blood lactate levels were measured in the same experimental setting. Compared with their respective controls, both the plain-dwelling (CL) and high-altitude–adapted (HL) groups exhibited significant lactate elevation following LPS injection (Figs. 1a–b). Intriguingly, the HL group showed a greater rise in lactate yet milder tissue injury. Histological analysis confirmed less mucosal edema, reduced crypt distortion, and lower pathological scores in HL rats than in CL rats (Figs. 1c–d). The microvillar structure remained largely intact, and the fluorescence intensity of ZO-1 and occludin was higher in the HL group (Figs. 1e–g). Serum TNF-α levels were also lower in HL rats, whereas the increase in MDA was less pronounced compared with the CL group (Figs. 1 h–i). Collectively, these findings indicate that high-altitude hypoxic adaptation decouples lactate elevation from tissue injury, suggesting that the lactate surge represents adaptive metabolic mobilization rather than simple hypoperfusion.

This observation conceptually complements the work by You et al., [1] implying that lactate can act as a reparative metabolic signal when the host is preconditioned by hypoxia. Chronic hypoxic exposure shifts cellular metabolism toward glycolysis, leading to endogenous lactate accumulation and stabilization of HIF-1α, a transcriptional activator of multiple epithelial growth factors. In our transcriptomic analysis, Areg (amphiregulin) and Ereg (epiregulin)—two pivotal EGFR ligands mediating epithelial restitution—were significantly upregulated in the HL group. KEGG and GO enrichment analyses further revealed activation of extracellular-region and inflammatory bowel disease–related pathways, involving Areg, Il22, Il17f, Ifng, and Pcsk9 (Figs. 1j–l). Consistent with prior reports emphasizing the key role of the Areg/EGFR signaling pathway in epithelial repair [3, 4], these results suggest enhanced epithelial regeneration and immune-barrier remodeling under HA conditions.

Integrating these data, we propose a lactate–Areg/EGFR regulatory axis as a potential molecular link between metabolic adaptation and mucosal protection. Elevated lactate may induce Areg/Ereg expression by stabilizing HIF-1α [5] and/or activating the G-protein–coupled receptor GPR81, both converging on the EGFR–ERK pathway to drive epithelial proliferation and tight-junction repair. This metabolic–signaling crosstalk explains why HL rats, despite higher lactate levels, exhibited improved barrier morphology and reduced inflammation. In this context, lactate no longer serves merely as a marker of hypoperfusion but as a metabolic signal encoding epithelial repair.

Our interpretation aligns with the emerging concept of metabolic conditioning in critical illness. High-altitude exposure represents a natural model of long-term hypoxic preconditioning that enhances mitochondrial resilience, redox balance, and autophagic capacity. The increased tolerance to transient lactatemia may reflect a reprogrammed metabolic–repair loopα.

Future studies should directly quantify intestinal tissue lactate and the expression of monocarboxylate transporters to validate this hypothetical axis. Interventions mimicking hypoxic preconditioning or modulating lactate signaling may reproduce similar protective effects at sea level. Such an approach could reconcile two seemingly paradoxical observations in sepsis: systemic lactate elevation accompanied by improved outcomes when metabolic adaptation is preserved.

Our study has several limitations. Firstly, we employed an intraperitoneal LPS model rather than a CLP model. Secondly, lactate measurements were restricted to arterial blood; therefore, although we observed an elevation in lactate levels associated with intestinal barrier repair and hypothesized that this lactate increase reflects the repair process rather than serving as a mere marker of injury, we lack exogenous lactate intervention experiments to validate the causal relationship underlying this hypothesis. Additionally, the concurrent upregulation of Areg/Ereg provides a plausible molecular mechanism explaining the link between lactate kinetics and epithelial resilience.

In summary, integrating the findings of You et al. [1] with our results provides a unified perspective: lactate is a context-dependent signaling molecule. In hypoxia-adapted hosts, lactate accumulation activates the Areg/EGFR pathway, promoting mucosal repair and maintaining intestinal homeostasis during sepsis. Recognizing this dual role of lactate may help design metabolic or preconditioning-based strategies to restore gut barrier function in critically ill patients.

**Fig. 1:**
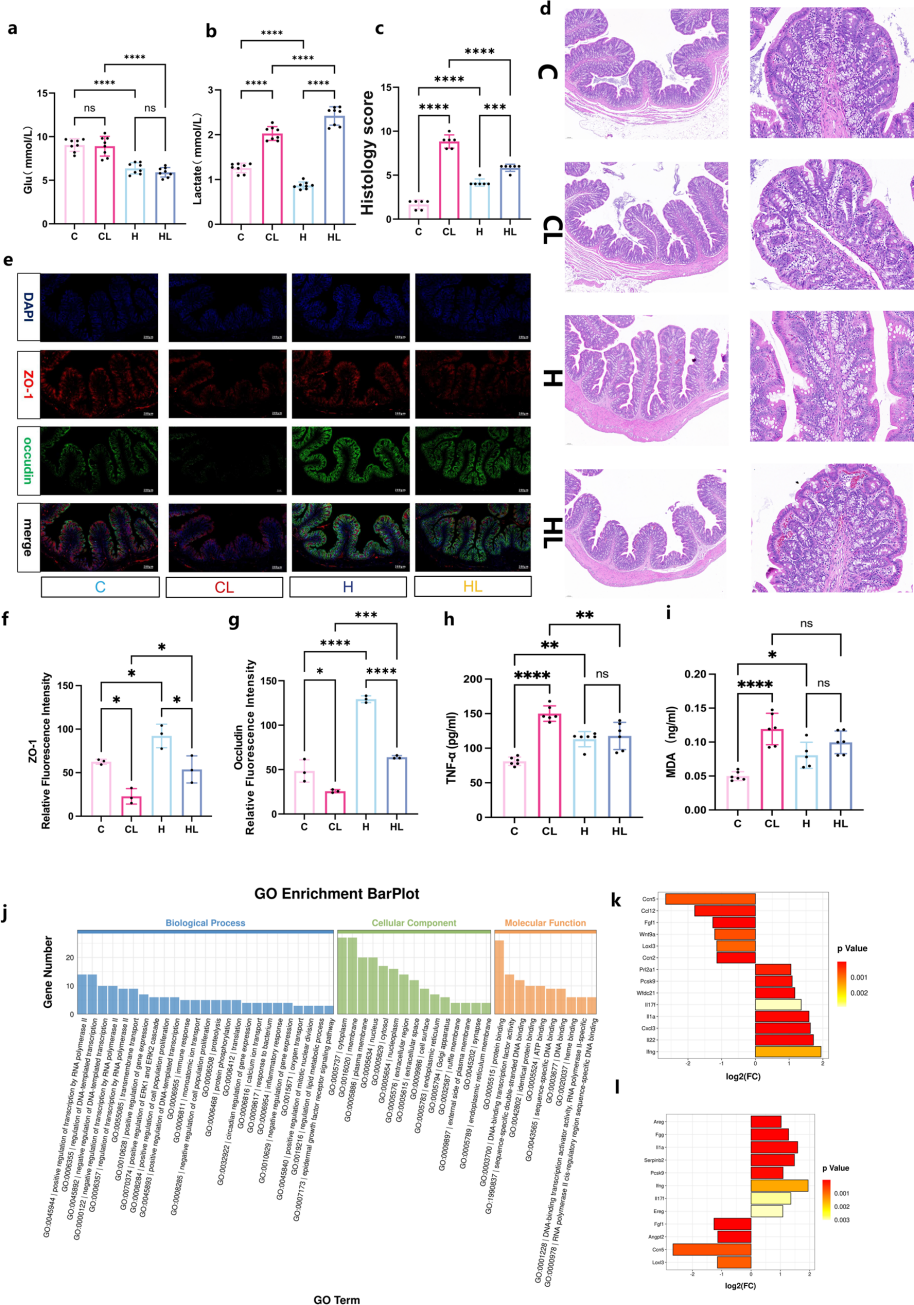
(**a**) Blood glucose levels in blood gas. (**b**) Lactate levels in blood gas. (**c**) Quantitative histopathological evaluation of colonic injury. (**d**) H&E-stained sections of colonic tissue. Scale bar: left: 0.1 mm; right: 0.02 mm. (**e**) Representative immunofluorescence images showing the expression of ZO-1 and occludin in the colon. Scale bar = 200 μm. (**f**) Quantification of relative fluorescence intensity for ZO-1 in colonic tissues. (**g**) Quantification of relative fluorescence intensity for occludin in colonic tissues. (**h**-**i**) Serum concentrations of MDA and TNF-α. (**j**) GO enrichment analysis of DEGs (Differentially Expressed Genes). (**k**) Bar chart showing DEGs in the ‘Extracellular Region’ pathway. (**l**) Bar chart showing DEGs in the ‘Extracellular Space’ pathway.

## Data Availability

The RNA-seq raw data are archived in the Genome Sequence Archive (GSA) at the National Genomics Data Center, China National Center for Bioinformation (Project ID: CRA025599; https://ngdc.cncb.ac.cn/search/).Other data are available from the corresponding author upon request.
